# Ocean warming and species range shifts affect rates of ecosystem functioning by altering consumer–resource interactions

**DOI:** 10.1002/ecy.3341

**Published:** 2021-04-30

**Authors:** Abby R. Gilson, Dan A. Smale, Nessa O’Connor

**Affiliations:** ^1^ School of Biological Sciences Institute of Global Food Security Queen’s University Belfast 1‐33 Chlorine Gardens Belfast BT9 5AJ UK; ^2^ Marine Biological Association of the UK Citadel Hill Plymouth PL1 2PB UK; ^3^ School of Natural Sciences Trinity College Dublin Dublin 2 Ireland; ^4^ Present address: Department of Zoology and Entomology Rhodes University PO Box 94 Grahamstown 6140 South Africa.

**Keywords:** climate change, community reconfiguration, detritivores, kelp forests, coastal marine ecosystems, ecosystem functioning

## Abstract

Recent warming trends have driven widespread changes in the performance and distribution of species in many regions, with consequent shifts in assemblage structure and ecosystem functioning. However, as responses to warming vary across species and regions, novel communities are emerging, particularly where warm‐affinity range‐expanding species have rapidly colonized communities still dominated by cold‐affinity species. Such community reconfiguration may alter core ecosystem processes, such as productivity or nutrient cycling, yet it remains unclear whether novel communities function similarly to those they have replaced, and how continued warming will alter functioning in the near future. Using simplified kelp forest communities as a model system, we compared rates of respiration, consumption and secondary productivity between current cold‐affinity and future warm‐affinity kelp assemblages under both present‐day temperatures and near‐future warming in a series of mesocosm experiments. Overall, respiration rates of gastropods and amphipods increased with warming but did not differ between cold and warm affinity kelp assemblages. Consumption rates of three consumers (urchin, gastropod and amphipod) differed between kelp assemblages but only amphipod consumption rates increased with warming. A diet derived from warm‐affinity kelp assemblages led to a decrease in growth and biomass of urchins, whereas the response of other consumers was variable depending on temperature treatment. These results suggest that climate‐driven changes in assemblage structure of primary producers will alter per capita rates of ecosystem functioning, and that specific responses may vary in complex and unpredictable ways, with some mediated by warming more than others. Understanding how differences in life history and functional traits of dominant species will affect ecological interactions and, in turn, important ecosystem processes is crucial to understanding the wider implications of climate‐driven community reconfiguration.

## Introduction

Anthropogenic climate change has led to a redistribution of species at the global scale, with consequent shifts in assemblage structure and ecological functioning in turn threatening the provision of valuable ecosystem services (Hoegh‐Guldberg and Bruno 2010, Pecl et al. 2017, Smale et al. 2019). In the marine realm, many species’ ranges have shifted poleward in response to warming (Pinsky et al. 2013, Poloczanska et al. 2013), with range shifts predicted to accelerate through the coming decades (Burrows et al. 2014, Jones and Cheung 2015, García Molinos et al. 2016). However, species’ distributions are not shifting in unison, as different species and even populations exhibit varying responses to warming, with mismatches in range shifts creating novel communities (Sorte et al. 2010, Walther 2010). In particular, an influx of warm‐water affinity species at higher latitudes is altering ecological interactions, driving species replacements and creating novel communities (Hawkins et al. 2008, Vergés et al. 2014, Wernberg et al. 2016, Frainer et al. 2017).

Community reconfiguration can have both direct and indirect consequences, such as reductions or increases in local species richness, altered carbon and nutrient cycles, and changes to food web structure and functioning (Dornelas et al. 2014, Lord and Whitlatch 2015, Smale et al. 2015, García Molinos et al. 2016, Pessarrodona et al. 2018, Blowes et al. 2019, Nielsen et al. 2019). Although some studies have examined the effects of replacement of cold‐water with warm‐water affinity species on local biodiversity (e.g., Teagle and Smale 2018), few have explored the consequences of species replacements for rates of ecosystem functioning (Mooney et al. 2009). Given that warm and cold‐water species differ in their phenology and functional traits (Smale et al. 2013, Wernberg et al. 2019, Pessarrodona et al. 2018*a*, *b*), there is a pressing need to examine the impacts of climate‐driven species replacements on core ecological processes.

Kelps (species of the order Laminariales) are large canopy‐forming seaweeds, which are distributed across ~25% of the world’s coastlines, primarily in temperate and subpolar regions (Wernberg et al. 2019). Kelps typically exhibit high levels of primary production, support increased secondary production and promote local biodiversity by providing biogenic habitat and modifying environmental conditions (Steneck et al. 2002, Smale et al. 2013). As the distribution of kelp species is strongly constrained by temperature, recent warming trends have been linked with changes in ecological performance, population structure, and geographical ranges (Smale 2020). In particular, populations of several warm‐adapted species have expanded poleward at their leading range edge and, conversely, several cold‐adapted species have contracted at the trailing range edge (Hiscock et al. 2004, Raybaud et al. 2013, Smale et al. 2013, Wernberg et al. 2019).

In some regions, disparity in rates of range shifts has led to the creation of novel communities, such as in southwest United Kingdom (UK), where the warm‐adapted kelp, *Laminaria ochroleuca*, has proliferated in recent decades in response to rapid warming and now coexists with the cold‐adapted assemblage dominants *L. hyperborea* and *L. digitata* (Smale et al. 2015, Hargrave et al. 2017). Northern populations of the Lusitanian “pseudo” kelp *Sacchoriza polyschides* (a kelp‐like species belonging to the order Tilopteridales) have also shown a shift toward increased abundance (Mieszkowska et al. 2006, Yesson et al. 2015, Brodie et al. 2014). Despite clear differences in the functional traits of these species, the wider implications of climate‐driven species replacements for the functioning of these ecosystems remain unresolved (but see Pessarrodona et al. 2018a). As kelp assemblages in the UK and Ireland comprise a mix of both warm and cold‐adapted species (Smale et al. 2013, Schoenrock et al. 2019), they provide a useful model system for examining indirect effects of ocean warming on ecological pattern and process. Although interspecific variability in consumption and degradation rates are poorly resolved, work to date suggests that warm‐adapted species such as *L. ochroleuca* are consumed more readily and decompose more quickly than their cold‐adapted counterparts (Hargrave et al. 2017, Pessarrodona et al. 2018*a*, *b*), perhaps due to lower C:N ratios (greater nutritional value) and concentrations of chemical defense compounds (Hargrave et al. 2017, Epstein et al. 2019).

It is estimated that >80% of kelp‐derived production enters the food‐web through detrital pathways (Krumhansl and Scheibling 2011). These subsidies influence energy and nutrient transfer, consumer dynamics and food web stability, all of which can depend on the magnitude, composition, and timing of the subsidy (Moore et al. 2004). Specifically, detrital subsidies can have significant effects on consumer–resource interactions, altering the dynamics and strengths of interactions in recipient food webs (Knight et al. 2005). In addition, the magnitude and direction of these effects may be temperature dependent, which affects rates of functioning for both consumer and resource species (Traill et al. 2010, Kordas et al. 2011). Current knowledge is limited to studies of effects of temperature on species interactions, which are generally restricted to direct consumption of primary producers (e.g., (O’Connor et al. 2009, Carr and Bruno 2013, Mrowicki and O’Connor 2015, Vye et al. 2015), with little known regarding subsidized food webs (i.e., by allochthonous detritus) where consumer populations are not dynamically coupled with primary production within the system (Greig et al. 2012).

The aim of this study was to disentangle the direct effects of increased temperature from the indirect effects of climate‐driven shifts in primary producer assemblage composition on rates of consumption and secondary productivity in a model kelp forest community. We tested empirically for differences in rates of consumption and secondary productivity, between current (cold water affinity) and predicted (warm water affinity) kelp assemblages, under current (ambient) and predicted (+2°C) future oceanic conditions in the northeast Atlantic region. Experiments were carried out using three common kelp consumers (the urchin *Echinus esculentus*, gastropod *Steromphala umbilicalis* [previously *Gibbula umbilicalis*], and amphipod *Gammarus* spp.) that are widely distributed throughout the northeast Atlantic and are expected to be dominant species in future communities in this region. We tested for direct effects of increased temperature on rates of respiration and consumption and secondary production of these three consumers, in the presence of current dominant kelp species (*Saccharina latissima* and *Laminaria digitata*) and compared them to those in the presence of predicted dominant kelp species (*Laminaria ochroleuca* and *Sacchoriza polyschides*). Specifically, three hypotheses were tested: (1) Warm affinity kelp species (*L. ochroleuca* and *S. polyschides*) will be consumed faster (by all three consumers) than cold‐water kelp (*S. latissima* and *L. digitata*), driven by their greater nutritional value and lower concentrations of chemical defenses. (2) Respiration and consumption rates will be greater in predicted warmer conditions for all three consumers owing to increased metabolism. And (3) synergistic effects of warming and shifts of assemblage structure will lead to overall greater rates of each of these ecological processes.

## Materials and Methods

### Experimental design and set‐up

Experimental kelp communities were assembled in each mesocosm and were designed to mimic local shallow subtidal algal‐dominated communities, which are representative of typical temperate reefs of the UK and Ireland. As the majority of this kelp‐derived production enters the food‐web through detrital pathways, we selected consumers that are largely detritivorous to capture detritivore–subsidy relationships (in contrast to previous studies of algal‐grazer interactions that have focused on herbivores, e.g., Mrowicki and O’Connor [2015]). Three experiments were conducted, with each adopting a different model consumer: the urchin, *Echinus esculentus*; the gastropod, *Steromphala umbilicalis* (previously *Gibbula umbilicalis*); and the amphipod, *Gammarus* spp. All consumer species are widely distributed throughout the northeast Atlantic and were chosen based on their common abundance in intertidal and subtidal kelp communities in this region and their key role as detritivores (Crothers 2001, Fredriksen 2003, Norderhaug et al. 2003, Kelly 2005, de Bettignies et al. 2020). These consumers are not range edge populations and no evidence to date suggests future climate‐driven range expansion or contraction in the UK and Ireland. Rather, they were selected from a limited set of kelp‐consumers that are likely to coexist with range‐expanding warm‐temperate kelps such as *L. ochroleuca* and *S. polyschides*, thus creating novel consumer–resource interactions (Smale et al. 2015, 2016, 2020, Pessarrodona et al. 2018*a*). In addition, species such as *E. esculentus* are key components in structuring kelp‐dominated systems in other regions and it is possible that their importance in the UK and Ireland will increase under future ocean warming and community reconfiguration (Sjøtun et al. 2006, Norderhaug and Christie 2009, Filbee‐Dexter and Scheibling 2014, Rinde et al. 2014, Leclerc et al. 2015). Although care was taken to ensure the selection of *G. locusta*, it is possible that other gammarid species may have been present and we, therefore, refer to these consumers as *Gammarus* spp. Other intertidal gammarids can be confused with *G. locusta* but are known to occupy different habitats, particularly more brackish waters than those sampled for this experiment (Costello et al. 1989). Individuals for the current study were collected specifically below *Ascophyllum nodosum* canopies in a fully marine environment, which is thought to be the preferred habitat of *G. locusta* (Pavia and Carr 1999).

Experiments were conducted using an outdoor flow‐through mesocosm system at Queen’s University Belfast Marine Laboratory, Portaferry, Northern Ireland. Experiments had a fully factorial design with two factors: (1) kelp assemblage composition (fixed, two levels: warm‐affinity species that are predicted to proliferate and extend their ranges poleward into cooler waters of the northeast Atlantic [a mixture of *Laminaria ochroleuca* and *Sacchoriza polyschides*; Hargrave et al. 2017] and typical colder‐affinity species that currently dominate low shore/shallow subtidal rocky habitats in cooler waters of the northeast Atlantic [a mixture of *Saccharina latissima* and *Laminaria digitata*; Simkanin et al. 2005); and (2) water temperature (fixed, two levels: ambient, +2ᵒC). In each experiment, warmed treatments were maintained at 2ºC above ambient temperature, which represents predicted sea surface temperature increase in the northeast Atlantic by 2090 (relative to 1990; Philippart et al. 2011) and is currently observed during short term extreme warming events in the region (Joint and Smale 2017). Ambient temperatures were 13.9° ± 1°C, 14.4° ± 0.5°C, and 12.9° ± 1.3°C for experiment 1, 2, and 3, respectively (Appendix S1: Table S1), Given that our consumer species are not range edge populations and can be distributed further south than the location of this study, warming treatments were not expected to exceed thermal thresholds but to increase consumer performance based on thermal tolerance curves and metabolism.

The mesocosm platform was supplied with sand‐filtered seawater directly from the adjacent Strangford Lough. The exact set‐up and duration of each experiment differed slightly to be representative and appropriate for the different consumers (see Appendix S1: Table S2 for more detail). Briefly, for *E. esculentus* a total of five replicate mesocosms were used for each treatment combination, whereas eight smaller replicates were used for *S. umbilicalis* and *Gammarus* spp. For *E. esculentus*, one individual was held in each mesocosm, whereas four and 10 individuals were held in each tank for *S. umbilicalis* and *Gammarus* spp., respectively. In addition, “no consumer” controls were established for each treatment combination, to quantify changes in macroalgal biomass independent from effects of consumers (Appendix S1: Table S2).

For *E. esculentus*, dump buckets were used to simulate wave action on rocky shores (approximate mean flow‐through rate, 4 L/minute). For *S. umbilicalis* and *Gammarus* spp., water flow was achieved using large buckets (40 L) containing eight smaller flow‐through hoses, with two hoses placed inside each mesocosm (approximate flow‐through rate, 1.5 L/minute). Heated temperature conditions were created using aquarium heaters (Elite Submersible 300 W, Hagen Inc., Mansfield, Massachusetts, USA) and temperature was estimated twice daily within each mesocosm for the duration of the experiment using a digital aquarium thermometer (Marina Aqua‐Minder, Hagen Inc.).

### Sample collection

Following collection from a nearby rocky shore (Walter Shore, County Down, Northern Ireland, UK; 54.382º N, 5.553º W), consumers were acclimatized to the mesocosm conditions for one week. Size classes for each species, *E. esculentus* (80–100 mm), *S. umbilicalis* (9–13 mm), and *Gammarus* spp. (3–5 mm) were selected to avoid collection of juveniles but allow scope for growth during the experiment. All consumers were fed ad libitum on macroalgal material found at their collection site before being starved for 48 h prior to the start of the experiment to empty the gut of any remaining food items and standardize initial nutritional state (Moulin et al. 2015). Three common kelp species (*L. digitata*, *S. latissima*, and *S. polyschides*) were collected prior to the experiment from local rocky shores (Knockinelder 54.384º N, 5.473º W and Walter Shore, County Down). A fourth range extending species that is not found locally, *L. ochroleuca*, was collected from Plymouth Sound, southwest UK (50.3606º N, 4.1625º W) and transported fresh in freezer boxes containing ice packs to Portaferry. Macroalgal material was cut into 5‐cm fragments, blotted dry, weighed to two decimal places. and frozen.

### Consumption rates

To test for differences in consumption rates of all three consumers among all experimental treatments, detrital kelp biomass was quantified at the beginning and end of each experiment. As most kelp production is consumed as detritus, macroalgae were frozen prior to the experiment to ensure death and facilitate their consumption as detrital material. Every three days during the experiment, a known biomass of freshly thawed kelp was fed to each consumer (biomass of each kelp species fed to each consumer were as follows: *E. esculentus*, 10–11 g; *S. umbilicalis*, 5–6 g; *Gammarus* spp., 5–6 g), with the warm‐affinity treatments receiving a dose of both *S. polyschides* and *L. ochroleuca* and the cold‐affinity treatments receiving both *S. latissima* and *L. digitata*. Pilot studies were used to estimate sufficient kelp biomass required for feeding. At the end of day three, all remaining kelp material was collected, blotted and weighed before the next feed. To standardize food availability and quality (nutritional value) as well as any changes in chemical composition (and therefore palatability), only basal parts of the blade of each kelp species were added to each mesocosm. To control for autogenic changes in kelp during each feed, biomass loss in control treatments without consumers was averaged for each treatment. The amount of kelp consumed in each mesocosm was then corrected for autogenic changes using the mean value from the corresponding treatment. Where more than one consumer individual was present in the mesocosm, the amount of kelp material consumed was divided by the number of individuals (g dry mass/individual).

### Respiration rates

To test for differences in respiration rates of all consumers among treatments, oxygen consumption rates were quantified by comparing dissolved oxygen concentrations in water in all mesocosms after periods of artificially induced dark incubation using an optical probe (HACH IntelliCAL LDO101, LoveLand, Colorado, USA; Noël et al. 2010). Any remaining kelp material was first removed and initial oxygen concentrations were measured within each mesocosm before water flow was immediately stopped. Mesocosms were then covered with black polyethylene covers and incubated in darkness for a period of 3–12 h, after which another set of respiration measurements were taken. Incubation periods for each consumer varied to ensure a quantifiable change in oxygen concentration was achieved and respiration rates were calculated as O_2_ consumption per individual per hour (mg/L; Noël et al. 2010, White et al. 2018); incubations were conducted toward the end of the experimental period and lasted 3 h for *E. esculentus* and *S. umbilicalis* and 12 h for *Gammarus* spp. Mesocosm temperatures were not measured continuously throughout the incubation period but any changes in temperature during incubations were consistent across treatments as all mesocosms were kept under similar conditions. As we were interested in testing for differences among treatments and not absolute oxygen consumption rates, any indirect changes to dissolved oxygen concentrations via changes in temperature would not affect the results. In addition, we wanted to mimic natural fluctuations in temperature associated with changes in tidal state or weather.

### Secondary production

To test for differences in growth and biomass of each of the three consumers among all experimental treatments, total length to the nearest millimeter and total wet biomass to the nearest 0.001 g (*E*. *esculentus*, test diameter; *S. umbilicalis*, shell length; *Gammarus* spp., tip of the head to the pleotelson) for each individual were taken at the beginning and end of each experiment. At the beginning of the experiment, it was not possible to measure live gammarids accurately with nonintrusive methods, thus, *Gammarus* spp. was subsampled destructively (*n* = 30 from the same population) and measured at the start of the experiment. The resulting average length of the sub‐sample was used as a proxy for the mean length of individuals prior to the start of the experiment. Mean consumer biomass of individuals was 387 ± 3 g (mean ± SD) for *E. echinus*, 1 ± 0.003 g for *S. umbilicalis*, and 0.006 ± 0.0002 g for *Gammarus* spp. The gonad index of *E. esculentus* was estimated to test for any effects of different kelp composition on their reproductive capacity. At the end of the experiment, wet gonad biomass was calculated by dissecting out the gonads, blotting dry, and weighing to the nearest 0.01 g. The gonad index was then estimated using the ratio of gonad wet biomass to total wet biomass. To test for and characterize relationships between wet and dry biomass for each species, all consumers were dried at 60°C until constant biomass was obtained to estimate dry biomass and then placed in a muffle furnace at 450°C for 3 h to obtain ash‐free dry mass.

### Data analysis

General linear models were used to test for differences between cold‐affinity and warm‐affinity kelp assemblages and temperature on consumption (of kelp mixtures and per individual kelp species) and respiration rates and secondary productivity (length and wet or ash‐free dry biomass) of three consumers (the urchin *Echinus esculentus*, gastropod *Steromphala umbilicalis*, or amphipod *Gammarus* spp.). All models included an interaction term to test for synergistic effects of increased temperature and different kelp assemblages but when not significant, interactions were removed and the model rerun with main terms only. If model assumptions were met, ANOVA was used to obtain *P* and *F* values (package car; Fox and Weisberg 2011). Where *P* values were significant, Tukey HSD adjusted pairwise comparisons using least‐square means were used for post hoc comparisons (package lsmeans; Lenth 2018). Residuals were visually inspected and QQ plots were used to check assumptions of normality and homogeneity of variance (Zuur et al. 2009). Where residuals did not meet model assumptions, data were log‐transformed and the model rerun. All analyses were conducted using R (R Development Core Team 2016).

## Results

### Consumption rates

**Fig. 1:**
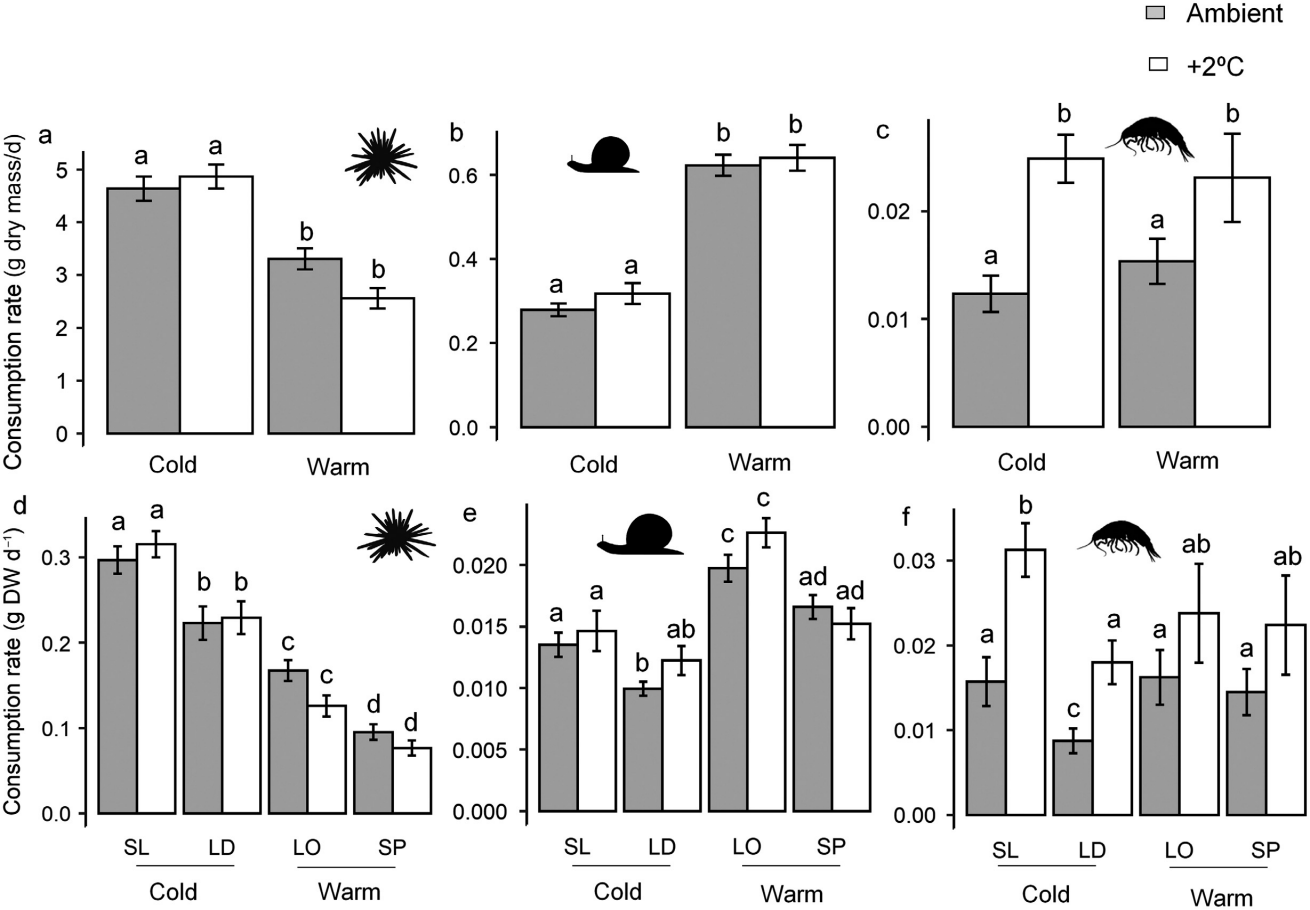
Consumption rates (mean ± SE) of cold and warm‐water affinity kelp assemblages (g dry mass/d) under different warming treatments (ambient and +2ºC) by (a, d) *Echinus esculentus*; (b, e) *Steromphala umbilicalis*; and (c, f) *Gammarus* spp. Kelp species are SL, *Saccharina latissima*; LD, *Laminaria digitata*; LO, *Laminaria ochroleuca*; and SP, *Saccorhiza polyschides*. Lowercase letters represent groups of means that are statistically indistinguishable (*P* < 0.05) between kelp assemblages and individual kelp species based on post hoc tests.

Consumption rates of *E. esculentus* (*F*_1, 893_ = 193.78; *P* < 0.001) and *S. umbilicalis* (*F*_1, 562_ = 51.6471; *P* < 0.001) differed between kelp assemblage treatments and post hoc tests identified that *E. esculentus* consumed significantly more cold‐affinity kelp detritus and *S. umbilicalis* more warm‐affinity kelp detritus (Fig. 1). No significant effect of temperature on the consumption rates of *E. esculentus* and *S. umbilicalis* was recorded. Consumption rates of *Gammarus* spp. did not differ between kelp assemblage treatments but there was a significant effect of temperature (*F*_1, 216_ = 16.177; *P* < 0.001) and post hoc tests identified greater consumption rates in warmer conditions (Fig. 1).

### Respiration rates

**Fig. 2:**
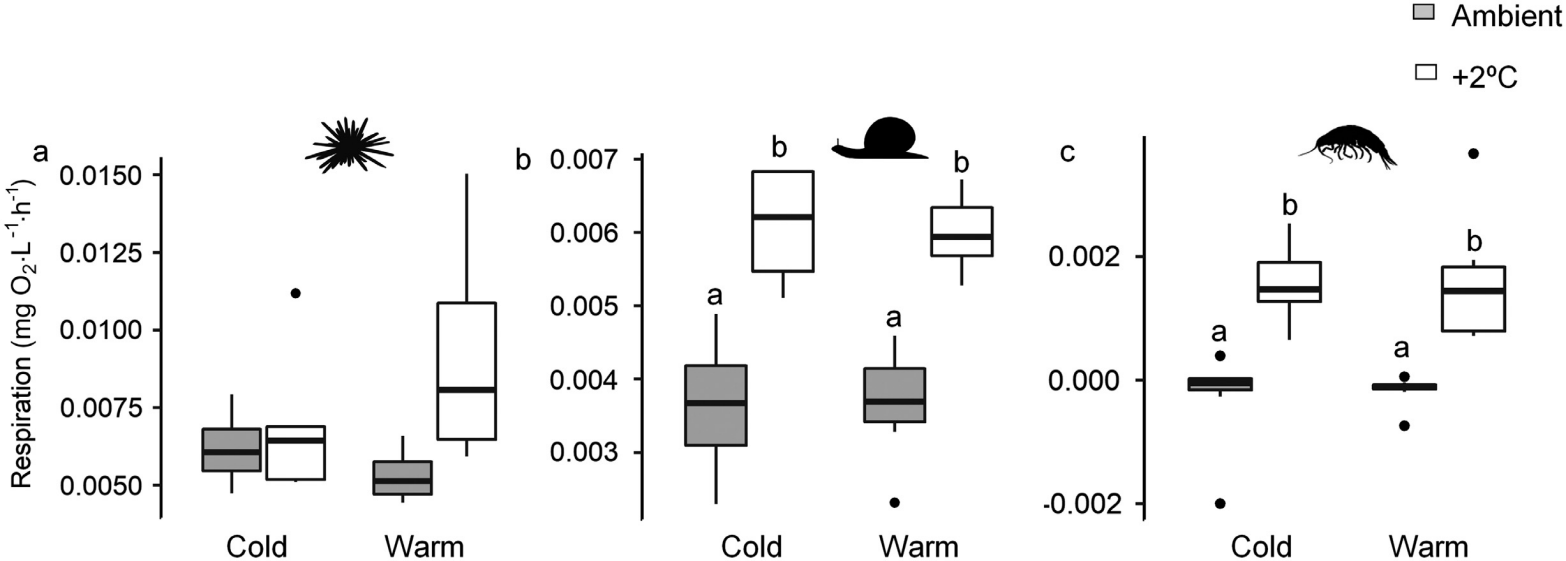
O_2_ consumption per individual per hour of three consumers: (a) *Echinus esculentus*, (b) *Steromphala umbilicalis*, and (c) *Gammarus* spp. fed different kelp assemblages (cold and warm affinity) under different warming treatments (ambient and + 2ºC). The upper and lower boundaries of each box represent the 75th and 25th percentiles, respectively, and the solid in the middle represents the median. Upper and lower whiskers represent the 90th and 10th percentiles, respectively. Lowercase letters represent groups of means that are statistically indistinguishable (*P* < 0.05) between kelp assemblages and individual kelp species based on post hoc tests.

No significant effect of kelp assemblage or temperature on the respiration rates of *E. esculentus* was identified, although rates appeared generally lower in warmed treatments (Fig. 2). There was a significant effect of temperature on the respiration rates of *S. umbilicalis* (*F*_1,28_ = 83.823; *P* < 0.001) and *Gammarus* spp. (*F*_1,29_ = 72.111; *P* < 0.05), with higher rates in warmer conditions when fed either kelp assemblage, but no significant effect of kelp assemblage (Fig. 2).

### Secondary production

**Fig. 3:**
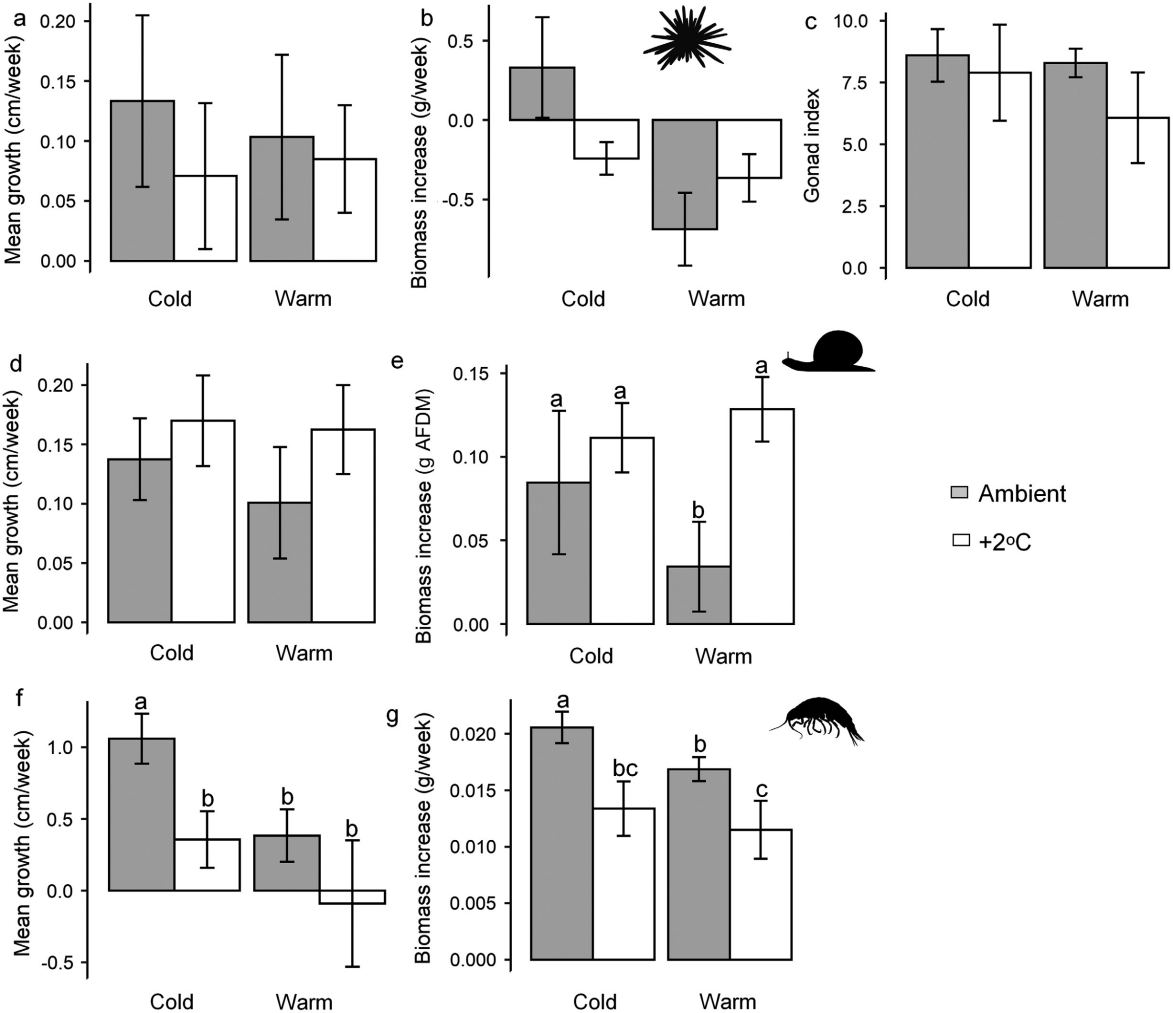
(a) Growth rates, (b) change in biomass, and (c) gonad index of *Echinus esculentus*, (d) growth rates and (e) change in ash‐free biomass (AFDM) of *Steromphala umbilicalis* and (f, g) *Gammarus* spp. fed different kelp assemblages (cold and warm affinity) under different warming treatments (ambient and +2ºC) over differing experimental periods (*E. esculentus* 12 weeks, *S. umbilicalis* 4 weeks, *Gammarus* spp. 2 weeks). Values are means ± SE; *n* = 5.

Variability between individuals was high, and we did not identify any significant effects of temperature or kelp assemblage on the growth, biomass and gonad index of *E. esculentus* (Fig. 3). There was a slight tendency toward reduced secondary production in warmed treatments, but this was not statistically significant (*P* > 0.05). High variability meant there was also no effect of temperature on the growth of *S. umbilicalis*, but there was a general trend toward increased secondary production in warmed treatments (Fig. 3). Ash‐free dry biomass differed between temperature treatments (*F*_1,26_ = 4.168; *P* = 0.05) and post hoc tests identified greater biomass in warmed treatments when fed warm‐affinity kelp detritus (Fig. 3). Growth of *Gammarus* spp. differed between temperature (*F*_1,26_ = 5.896; *P* = 0.02) and kelp assemblage (*F*_1,26_ = 5.136; *P* = 0.03) treatments. Although growth was consistently lower when fed warm‐affinity kelp detritus, post hoc tests identified that growth was only significantly lower in ambient treatments (Fig. 3). Ash‐free dry biomass of *Gammarus* spp. also differed between temperature treatments (*F*_1,26_ = 12.0086; *P* < 0.001) and post hoc tests identified consistently lower biomass in warmed conditions (Fig. 3).

## Discussion

Our study shows how indirect effects of warming (i.e., climate‐driven shifts in resource species composition) can interact with direct effects (i.e., increased temperature) to alter key rates of ecosystem functioning and highlights how responses are not uniform across consumer–resource interactions. For example, in warmed treatments respiration rates of *S. umbilicalis* and *Gammarus* spp. increased, but not *E. esculentus*, and only the consumption rates of *Gammarus* spp. increased when warmed. The consumption rates of cold‐ and warm‐water kelp assemblages differed among all consumers. *E. esculentus* consumed significantly more of the current cold‐adapted dominant kelp species (*S. latissima* and *L. digitata*) and *S. umbilicalis* consumed more of the predicted future dominant kelp species (*L. ochroleuca* and *S. polyschides*), but *Gammarus* spp. showed no preference. Similarly, effects of experimental treatments on secondary productivity were inconsistent, as two consumers showed tentative signs of reduced growth under warmer conditions, while *S. umbilicalis* appeared to increase growth rates and ash‐free biomass in response to warming. Secondary productivity did not differ between cold and warm affinity kelp assemblages for any consumer. Our hypothesis that direct and indirect effects of climate change would interact synergistically to increase rates of functioning was, therefore, only partially supported and, instead, responses were complex, species‐specific, and less predictable.

The temperature dependence of respiration rates in *S. umbilicalis* and *Gammarus* spp. is in line with current metabolic theory (Pӧrtner 2010). Under warming scenarios, these significant increases in per capita rates of metabolism drive increases in community respiration and lead to significant reductions of gross and net assemblage productivity (White et al. 2018). These consequences, however, will be mediated by shifts in the properties of resources and the body size of consumers associated with increased temperatures (Binzer et al. 2016). Given the temperature dependence of trophic cascades, these small changes at the individual level can have significant consequences for ecosystem‐scale processes (Hansson et al. 2013, Svensson et al. 2017). The lack of a temperature effect identified in *E. esculentus* may reflect an ability to acclimate to higher temperatures, particularly when temperatures do not exceed seasonal temperature cycles and maximum summer means found in their habitats (Siikavuopio et al. 2008). Acclimation has been identified in multiple species of sea urchin and has been shown to occur within the time‐frame of the current experiment (Harianto et al. 2018, Rich et al. 2018). Further experiments in which respiration rates are monitored periodically throughout the experiment are needed to identify potential differences in acute and adaptive responses.

Considering there was no effect of temperature on the respiration rates of *E. esculentus*, the lack of temperature effect on consumption rates is not surprising as there would be no significant increase in energy demand. *E. esculentus* did, however, consume significantly greater quantities of cold‐affinity kelps despite their lower nutritional value and greater concentrations of chemical defenses compared to their predicted warm‐affinity counterparts (Hargrave et al. 2017, Epstein et al. 2019). Sea urchins and macroalgae have a strong evolutionary history, coevolving with the macroalgal species present within their habitat over time (Vadas 1977). As such, urchins have strong preferences in food choice experiments and greater consumption of macroalgal species that is naturally abundant in their habitat has been reported for many species (Vadas 1977, Tomas et al. 2011, Foster et al. 2015). It has been suggested that the ability to detect particular chemical cues or the development of specific digestive enzymes that are compatible with specific macroalgal species may contribute to the high affinity of sea urchins to their most preferred food choice (Lawrence et al. 2013). Populations of *E. esculentus* in the UK and Ireland have no evolutionary relationship with range‐expanding warm‐affinity kelps and may not be adapted to find, consume, and digest range‐expanding kelps such as *Laminaria ochroleuca*. Although *S. polyschides* is regularly found within the habitat of *E. esculentus,* consumption rates were very low and may reflect the pseudo‐annual life history and patchy distribution of this species offering an inconsistent food supply, with urchins choosing to consume perennial, cold‐water, kelp species that persist all year round. In other regions such as Norway, however, *E. esculentus* can play a crucial role in structuring kelp‐dominated systems (Sjøtun et al. 2006, Norderhaug and Christie 2009). Under future scenarios of community reconfiguration and ocean warming, *E. esculentus* may become a key component in controlling kelp forest structure, as has been reported for other species of urchin in the northeast Atlantic including *Strongylocentrotus droebachiensis* and *Paracentrotus lividus* (Skadsheim et al. 1995, Leinaas and Christie 1996, Trowbridge et al. 2011).

Despite an increase in the respiration rates of the gastropod *S. umbilicalis* at higher temperatures, warming did not increase consumption rates as hypothesized. *S. umbilicalis* is an intertidal gastropod and is frequently exposed to thermally stressful conditions during periods of emersion where substratum and air temperature can often exceed the upper physiological limits of many gastropod species (Chapperon et al. 2017). As a result, *S. umbilicalis* may have adapted or acquired mechanisms to withstand broader temperature ranges that exceed the temperatures used within the study. Other studies have shown that organisms inhabiting thermally variable environments may be less sensitive to changes in temperature (Yee and Murray 2004). Although the mechanisms behind this temperature tolerance are largely unknown, a study by Yee and Murray (2004) on the grazing gastropod *Tegula* spp. found that individuals inhabiting populations spanning the full latitudinal range of their distribution were more effective at repairing heat‐damaged proteins than those taken from smaller, more geographically restricted, populations. In addition, *S. umbilicalis* are not obligate kelp grazers and may have benefited from an increase in microalgal growth at higher temperatures, actively selecting microbial community consumption over kelp detritus and obscuring the effect of temperature on consumption rates (Sarmento et al. 2010). Greater consumer‐driven control is predicted to significantly alter plant‐herbivore interactions under warming scenarios (O’Connor 2009). If gastropods are able to acclimate to broader temperature ranges or metabolic demand only drives greater consumption during peak annual temperatures, macroalgal growth may be compensated for during periods of lower grazing pressure.

Crucially, *S. umbilicalis* consumed significantly greater quantities of warm‐affinity kelps in both temperature treatments, perhaps due to the greater nutritional content and reduced chemical defenses of some warm‐affinity kelp species (Hargrave et al. 2017, Epstein et al. 2019). Food preference and selectivity have been shown to correlate with nutritional content, particularly nitrogen, in a number of marine grazers (Van Alstyne and Houser 2003). Avoidance or low feeding rates of macroalgae have been attributed to high concentrations of secondary metabolites, such as polyphenols, that provide a chemical defense against herbivore grazing (Granado and Caballero 2001). This is in line with comparative studies (on fresh kelp) undertaken in the English Channel in which *Laminaria ochroleuca* has been found to support significantly higher abundances of *Patella pellucida* and *Steromphala* (formerly *Gibbula*) *cineraria* than its cold‐water counterparts *L. digitata* and *L. hyperborea* (Smale et al. 2015, Hargrave et al. 2017, Pessarrodona et al. 2018*a*). Greater consumption of warm‐affinity kelps in the future may lead to greater top‐down control by grazers, altering the way in which carbon flows through food web compartments. This may lead to far‐reaching ecological consequences given that herbivory is not recognized as a major driver of kelp population structure in Ireland and the UK where grazer densities are relatively low and over‐grazed barrens are the exception rather than the rule (Smale et al. 2013, Hargrave et al. 2017, Hereward et al. 2018).

The amphipod *Gammarus* spp. was the only consumer in our study for which consumption rates were directly affected by warming, consuming greater amounts of macroalgae at higher temperatures. Under warming scenarios, the critical role of amphipods in the cycling of organic matter may lead to greater rates of nutrient turnover, decreasing residence time of kelp detritus and leading to functionally faster systems. Given that warm‐affinity kelps have been shown to degrade an average of 6.5 times faster than cold‐water kelps, faster turnover rates of detritivores under future warming will further exacerbate the consequences of community reconfiguration (Pessarrodona et al. 2018a). It is possible, however, that the observed increase in consumption rates may be a short‐term response and that adaptation may occur in longer‐term experiments.

We identified no indication of preference of *Gammarus* spp. for either kelp assemblage. A substantial capacity for the consumption of taxonomically and morphologically diverse macroalgae and a lack of discrimination between macroalgal species has been widely reported for marine amphipod species (Taylor and Brown 2006). Food choice and feeding rates have been shown to positively, negatively, or not correlate with factors such as nutritional quality, chemical defenses, morphology, and toughness (Cruz‐Rivera and Hay 2001, Jormalainen et al. 2001, Van Alstyne et al. 2001, Duarte et al. 2011). The ability to utilize multiple food sources may explain how populations of marine amphipods are able to persist year‐round in high densities and inhabit broad ecological and environmental niche spaces (Cruz‐Rivera and Hay 2001, Taylor and Brown 2006). In smaller, less mobile mesograzers, it has also been suggested that macroalgal selection is based primarily on habitat quality and secondarily on its value as a food source (Cox and Murray 2006, Lasley‐Rasher et al. 2011). Although it is unlikely that community reconfiguration will affect the consumption of macroalgal detritus by amphipods, differences in the timing and quantity of detrital production between current and predicted kelps may affect food availability, driving changes in population densities and subsequent nutrient cycling.

Despite the significant increase in consumption rates at higher temperatures in our study, secondary biomass in *Gammarus* spp. was significantly reduced in warmed treatments. Gut traits pose constraints on the quantity of food that can be consumed at any one time, limiting acquisition of adequate nutrients required for an associated increase in metabolic demand (Cruz‐Rivera and Hay 2001). As a result, compensatory feeding is not always enough to compensate for negative effects on fitness (Cruz‐Rivera and Hay 2001, Duarte et al. 2011, 2014). The ubiquitous occurrence of amphipods (of the *Gammarus* genus and others) in high densities in most marine habitats underscores their critical role in marine food webs (Bruno and O’Connor 2005). As detritivores, they contribute significantly to coastal carbon and nutrient cycles through the recycling of organic matter and are a primary prey for a large number of benthic consumers (Nyssen et al. 2002, Dauby et al. 2003, Padovani et al. 2012). Changes to secondary biomass will have significant consequences for coastal food webs, slowing turnover rates of organic matter and reducing nutrient availability. In addition, reduced prey availability at lower trophic levels will propagate to higher trophic levels, reducing overall food web biomass, energy transfer efficiency and ultimately, food web stability (Dermott 2001).

In summary, our findings suggest that direct (i.e., increased temperature) and indirect (i.e., community reconfiguration) effects of ocean warming can alter per capita rates of ecosystem functioning, but that the strength and direction of these consequences will depend highly on consumer identity. We highlight how species relationships can change under predicted climate change scenarios and the importance of life history and properties of both consumers and resources. Compensatory mechanisms such as trait selection, however, may mediate the effects of temperature on the structure and functioning of ecological communities, maintaining key rates of ecosystem functioning. Understanding how these changes to species interactions at the individual level will translate to the population level and ultimately impact the functioning of our ecosystems is critical to predicting and managing the effects of a changing climate. The ability of ecological and evolutionary processes to moderate and maintain community‐level responses to environmental change must be the focus of future work.

## Supporting information

Appendix S1
