# Supplementary material for: Ocean warming and species range shifts affect rates of ecosystem functioning by altering consumer–resource interactions
Source: Ecology. 2021 Apr 30;102(5):e03341. doi: 10.1002/ecy.3341 (PMC11475527; doi:10.1002/ecy.3341)
Supplement: Supplementary file 1 — Appendix S1 [file ECY-102-e03341-s001.pdf]

Supporting Information. Gilson, A.R., D.A. Smale, and N. O'Connor. 2021. Ocean warming and species range shifts affect rates of ecosystem functioning by altering consumer–resource interactions. Ecology.

Appendix S1

Table S1. Mesocosm temperatures ± 1SD (°C) averaged across the experimental period for experimentally manipulated temperature treatments (fixed, two levels: ambient, heated) to test for effects of temperature on respiration and consumption rates and secondary production in a range of consumers (*Echinus esculentus*, *Gibbula umbilicalis* and *Gammarus* spp.).

Experiment	Ambient	Heated
E. esculentus	13.9 ± 1	15.9 ± 0.99
G. umbilicalis	14.4 ± 0.48	16.4 ± 0.68
Gammarus spp.	12.9 ± 1.3	15 ± 1.05

Table S2. Mesocosm experiments testing for the effects of differences in kelp community composition (cold-water affinity, *Laminaria digitata* and *Saccharina latissima* versus warm-water affinity, *Laminaria ochroleuca* and *Sacchoriza polyschides*) on rates of respiration, consumption and secondary production of three consumer species (the urchin, *Echinus esculentus*, gastropod, *Gibbula umbilicalis* and amphipod, *Gammarus* spp.). Each experimental treatment was designed to mimic consumer density at local sites. Controls were treatment combinations without consumers present to quantify natural macroalgal biomass breakdown/growth independent from effects of consumers. Mesocosm dimensions differ between experiments owing to different size mesocosms. L = length, W = width, H = height, D = diameter, V = volume.

**Experiment**	**Mesocosm dimensions**	**Individuals per mesocosm**	**Treatment replication (*n*)**	**Control replication (*n*)**	**Start date**	**Duration (weeks)**
E. esculentus	L 55.5 X W 35.5 X H 22<br>V 45 L	1	5	3	31/08/17	12
G. umbilicalis	H 26 X D 25<br>V 10 L	4	8	8	31/08/17	4
***Gammarus* spp.**	H 26 X D 25<br>V 10 L	10	8	8	02/11/17	2
